# Experimental brain infection with cysticercosis in sheep

**DOI:** 10.17843/rpmesp.2022.393.11039

**Published:** 2022-09-30

**Authors:** Katherine A. Sota, Javier A. Bustos, Manuela R. Verastegui, Luz Toribio, Nancy Chile, Noelia Angulo, Carla Cangalaya, Juan Calcina, Armando E. González, Robert H. Gilman, Héctor H. García

**Affiliations:** 1 Cysticercosis Unit, Instituto Nacional de Ciencias Neurológicas, Lima, Peru. Cysticercosis Unit, Instituto Nacional de Ciencias Neurológicas Lima Peru; 2 Global Health Center, Universidad Peruana Cayetano Heredia, Lima, Peru. Universidad Peruana Cayetano Heredia Global Health Center Universidad Peruana Cayetano Heredia Lima Peru; 3 Research and Development Laboratories, Universidad Peruana Cayetano Heredia, Lima, Peru. Universidad Peruana Cayetano Heredia Research and Development Laboratories Universidad Peruana Cayetano Heredia Lima Peru; 4 Escuela de Medicina Veterinaria, Universidad Nacional Mayor de San Marcos, Lima, Peru. Universidad Nacional Mayor de San Marcos Escuela de Medicina Veterinaria Universidad Nacional Mayor de San Marcos Lima Peru; 5 Department of International Health, Johns Hopkins Bloomberg School of Public Health, Baltimore, Maryland, US. Department of International Health Johns Hopkins Bloomberg School of Public Health Baltimore Maryland US

**Keywords:** Epilepsy, Neurocysticercosis, Sheep, *Taenia solium*, Cysticercosis

## Abstract

**Objective.:**

To explore the feasibility of developing a sheep model of neurocysticercosis (NCC) by intracranial infection with T. solium oncospheres.

**Materials and methods.:**

We carried out an experimental infection model of NCC in sheep. Approximately 10 T. solium oncospheres previously cultured for 30 days were inoculated intracranially into ten sheep. The oncospheres, in 0.1 mL of physiological saline, were injected into the parietal lobe through an 18-gauge needle.

**Results.:**

After three months, granulomas were found in two sheep. In a third sheep we identified a 5 mm diameter cyst in the right lateral ventricle and histological evaluation confirmed that the cyst corresponded to a T. solium larva. Immunohistochemistry with monoclonal antibodies directed against membrane components and excretory/secretory antigens of the T. solium cyst was also used to confirm the etiology of the found granulomas. One of them showed reactivity to the monoclonal antibodies used, thus confirming that it was a cysticercus.

**Conclusion.:**

This experiment is the proof of concept that it is possible to infect sheep with cysticercosis by intracranial inoculation.

## INTRODUCTION

Neurocysticercosis (NCC) is the infestation of the human nervous system by the larval stage of *T. solium*. It is a major public health problem in most developing countries, and is associated with significant neurological morbidity ^1^. Diagnosis of this disease is also increasing in industrialized countries due to migration from endemic areas, with approximately 2000 new cases diagnosed per year in the United States alone, where hospitalizations and associated costs attributable to NCC exceed the totals for malaria as well as all other neglected tropical diseases ^2^. In Peru, and in most endemic regions, about 30% of all epileptic syndromes appear to be attributable to NCC ^3^.

Humans often become infected with *T. solium* eggs by the fecal-oral route. In the intestine, intestinal fluid dissolves the egg coat, releasing and activating the oncosphere. Once activated, it can penetrate the intestinal wall. When it reaches the tissue through the bloodstream, usually the muscle or central nervous system, the oncosphere is established and the cysticercus develops. Subsequently, the parasite produces a variety of molecules that modulate the host immune response in order to prevent its destruction ^4^. Clinical manifestations depend on the organs affected. In humans, symptoms are mainly due to central nervous system involvement. Cysts located within the brain parenchyma (intraparenchymal NCC), usually degenerate in the following sequence: from viable cysts to inflamed cysts, to focal granulomatous tissue, and approximately 40% end with the formation of a calcified scar ^5^.

Many aspects of the disease, such as inflammation, immune response, the process of parasitic degeneration and calcification, the mechanisms leading to seizures and epilepsy, and the roles of antiparasitic and anti-inflammatory treatment, are still poorly studied, largely due to the lack of suitable animal models.

Naturally infected pigs have been used to study immunological and histopathological aspects ^6^ ^,^ ^7^, as well as to evaluate the pharmacokinetics, safety and efficacy of antiparasitic treatments ^8^ ^,^ ^9^. The swine model has also been used for experimental infection studies on this disease ^7^ ^,^ ^10^ ^,^ ^11^. However, experimental infection of the pig central nervous system (CNS) with *T. solium* by the oral route is difficult due to the variability in the efficacy of oral infection. Our group has developed an intracarotid oncosphere injection model that consistently produces NCC ^12^. However, the porcine model has disadvantages of the cost of purchasing and maintaining pigs for a long time and, most importantly, the scarcity of commercially available reagents for biomarker detection in pigs ^13^. Additionally, pigs rarely present clinically evident seizures.

Our group has also standardized an experimental infection model of cysticercosis in rat brain. In this model, activated *T. solium* oncospheres were injected intracranially and developed into brain cysts or metacestodes with characteristics identical to those observed in the natural host. Although this model does not follow the usual intestinal route of entry, reproducible infections occurred, similar to those observed in pigs and humans. Most infected rats (64%, n=42) developed cysticercus cysts in their brains. Infection was successful in 83% (10/12) of rats injected with 500 or 750 activated oncospheres, producing rats with one or more cysts. In addition, infected rats developed specific antibodies to *T. solium*, and circulating *T. solium* antigen could also be detected in serum and cerebrospinal fluid (CSF) using enzyme-linked immunosorbent assay (ELISA). This same model has been used by injecting the post-oncosphere form, already cultured *in vitro* for 15 days. In 2019, Palma *et al*. infected eight Holtzman rats intracranially with ten *T. solium* posoncospheres. Four months later, 63% (n=5) of these had intracranial infection with *T. solium* ^4^. However, these models have limitations. The rat is not the natural host of the disease because it has a short life span, which does not allow studying the chronicity of the disease and, due to the size of its brain, there could be an overestimation of the mass effect and the inflammation caused by the *T. solium* cyst ^13^.

Sheep have been used as animal models for various purposes when it comes to neurological conditions, including epilepsy, and non-neurological diseases. The sheep model is considered a suitable animal model for studying epilepsy because of its size, availability, and low maintenance cost. In addition, its anatomical arrangement facilitates the application of standard neurosurgical and neuroradiological techniques applied in humans, as well as the use of magnetic resonance imaging and other imaging methods ^14^ ^,^ ^15^.

This study was based on studies conducted to standardize the intracerebral infection model in rats with the aim of exploring the feasibility of developing an experimental infection model with NCC in sheep by intracranial infection with *T. solium* posoncospheres.

**KEY MESSAGES**

Motivation for the study: an appropriate animal model is necessary to study little known aspects of NCC and epilepsy, such as new antiepileptic treatments, vaccines, the cerebral inflammatory response, and its management, as well as the processes of calcification and epileptogenesis.

Main findings: granulomas were found in two sheep, and a third sheep presented a cyst in the right lateral ventricle.

Implications: this experiment is the proof of concept that it is possible to infect sheep with cysticercosis by intracranial inoculation, and an initial step to establish a new animal model capable of studying new approaches to this disease and the chronic effects of NCC and epilepsy.

## MATERIALS AND METHODS

### Animals

Ten healthy sheep between four and eight months of age, acquired from non-industrialized rural farms, were infected. The animals were checked by a veterinarian to confirm their good health status and transported to the large animal facility of the School of Veterinary Medicine of the Universidad Nacional Mayor de San Marcos in Lima. The sheep were housed in a 5x5 meter pen and kept on 12/12 hour light/dark cycles with a controlled average temperature of 16-18 ºC; mineralized salt and food rations were determined by age and water was provided *ad libitum*.

### Ethical Aspects

This protocol was carried out under the approval of the Institutional Ethics Committee for the Care and Use of Animals of the Universidad Peruana Cayetano Heredia.

### Preparation of posoncospheres

Based on previous protocols for experimental infection in rat brains ^13^, we carried out an experimental infection using *T. solium* posoncospheres. To obtain the posoncosphere stage, eggs were obtained from gravid proglottids of *T. solium* expelled after standard treatment of tapeworm carriers with niclosamide ^13^ and incubated in sodium hypochlorite (0.75%) for 10 min at 4 °C. Subsequently, the oncospheres were activated and then grown in a monolayer of HCT-8 culture cells for thirty days ^13^ ^,^ ^16^.

### Intracranial infection

**Figure 1:**
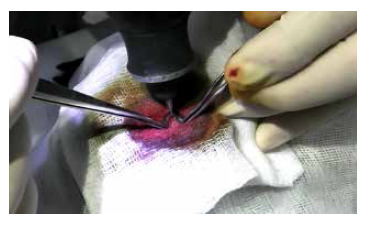
Procedure to make a small hole over the right parietal lobe.

Approximately ten *T. solium* posoncospheres were inoculated intracranially into each sheep. The animals were anesthetized and sedated using ketamine (Ket-A-10®), 20 mg/kg intramuscular (IM); xylazine hydrochloride (Dormi-Xyl®2), 0.3 mg/kg IM, and ketoprofen (Profenid®) 3 mg/kg IM. Under adequate aseptic conditions, a 2 cm incision was made in the skin of the right parietal area, and then a hole was made in the bone surface with a 2 mm diameter drill (Figure 1). Postoncospheres in 0.1 mL of physiological saline were injected into the parietal lobe with an 18-gauge needle. The 18-gauge needle has an inner diameter of 0.84 mm, which allows the passage of 30-day-old oncospheres, which have a diameter of approximately 0.5 mm. The needle was inserted approximately 2 cm deep to reach the parietal brain parenchyma. Five of the ten animals were immunosuppressed using 1 mg/kg/day of methylprednisolone for 3 days immediately after infection ^17^. In addition, they received ketoprofen 2 mg/kg/day IM for 3 days as postsurgical analgesic therapy.

The animals were euthanized under general anesthesia, 3 months after experimental infection. For this, we used an intramuscular combination of ketamine (20 mg/kg), and xylazine hydrochloride (2 mg/kg), IM. Subsequently, an overdose of sodium pentobarbital as a hypnotic and anticonvulsant (Halatal®) 120 mg/kg, also IM, was used. The whole brains were removed for examination and dissection. Abnormal structures were identified and stained with hematoxylin-eosin (H-E) for analysis. In addition, internal organs were carefully examined to identify concomitant parasitic infections.

### Immunohistochemistry

Immunohistochemistry (IHC) with moAbs directed against membrane components and excretory/secretory antigens of the T solium cyst was used to confirm the etiology of the lesions found ^18^.

Suspected lesions surrounded by tissue were placed in cassettes that were labeled and immersed in 10% paraformaldehyde buffer, then immersed in 70% ethanol for 24 to 48 h and, finally, embedded in kerosene according to standard procedures. Tissue sections were adhered to poly-L-lysine-impregnated slides, which were deparaffinized and rehydrated by immersion in xylol, ethanol (absolute, 96% and 70%). IHC was carried out by inactivating endogenous peroxidase with a dilution (1:5) of H_2_O_2_ for 30 min at room temperature and in the dark. For antigen retrieval, we used citrate solution at 95 °C for 8 min and allowed to cool. Then, tissue sections were washed three times with PBS buffered solution (pH 7.2) and to which we added blocking solution with 10% goat serum and 6% milk diluted in PBS-Tween 0.05% -Triton 0.1% for 1 h. The culture supernatants of anti-T solium TsW5/ Tsw11/ TsE3 moAbs ^18^ were diluted to the determined concentration in PBS-Tween 0.05% -Triton 0.1% and incubated at 4 °C overnight. Additionally, we placed positive (*T solium* cysts from pigs), negative (healthy sheep brain tissue), and technique (sheep and pig cysts without added moAb) controls. The slides were washed three times with 0.05% PBS-Tween and biotinylated IgG-conjugated anti-mouse secondary antibody diluted 1/700 in blocking solution with 10% pig serum and incubated for 30 min. The moAbs-antigen junctions were amplified with streptavidin-HRP for 30 min. Finally, the reaction was evidenced using a solution with the chromogen 3,3-diaminobenzidine (DAB); hematoxylin solution was added as tissue contrast, followed by dehydration with alcohols and xylol, then mounting was carried out.

## RESULTS

**Figure 2:**
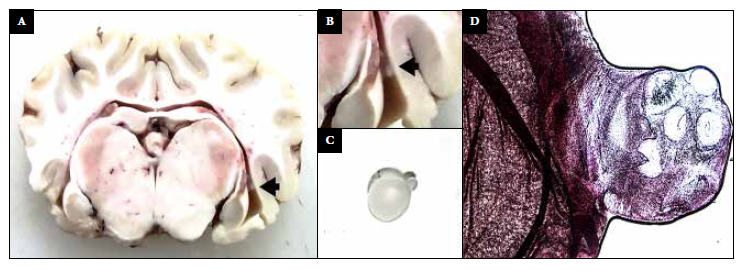
A-B: photograph of a sheep brain showing a ventricular cyst (black arrow). C: Ventricular cyst with evaginated scolex. D: H-E staining (40x) shows scolex, four suckers and a rostellum.

Intracranial inoculation with posoncospheres was carried out in ten sheep. There were no complications after the intervention and the animals showed no signs of pain according to the scale proposed by Guedes *et al*. ^19^. One hour after anesthesia was administered, the sheep gradually returned to normal behavior. Three of them died before the end of the 90-day follow-up. According to necropsy reports, two of them died due to intestinal obstruction by *Moniezia expansa*, and one due to intestinal bacterial infection. The remaining seven sheep, five from the immunosuppressed group and two from the non-immunosuppressed group, were euthanized 3 months after inoculation. The brains were extracted whole for pathological analysis. In four of them we did not find no structure suggestive of cysticercosis infection, in two sheep non-specific granulomatous lesions were found and in one a 5 mm diameter protruding ventricular cyst was identified in the right lateral ventricle. By direct microscopy and histological evaluation with H-E staining, the cyst was confirmed to correspond to a *T. solium* larva, a rostellum (crown of hooks) and four suckers were clearly identified (Figure 2). Microscopy of one of the granulomas also demonstrated remnants of parasitic tissue compatible with a cestode larva, and the other showed only a cluster of lymphocytes. These abnormal findings (two granulomas and one cyst) were found in three sheep from the immunosuppressed group.

All sheep showed other concomitant parasitic infections at necropsy. Eight were infected with *Moniezia expansa*, five with *Fasciola hepatica*, two with *Thysanosoma actinioides* and two with *Taenia hydatigena*. Six of them had multiparasitic infections.

### Immunohistochemistry

**Figure 3:**
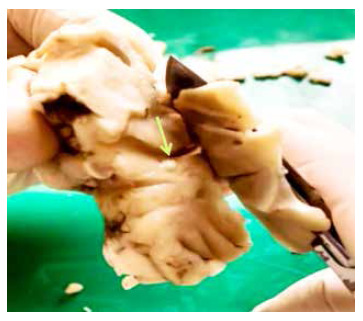
Photograph of a sheep brain showing a non-specific granuloma.

**Figure 4:**
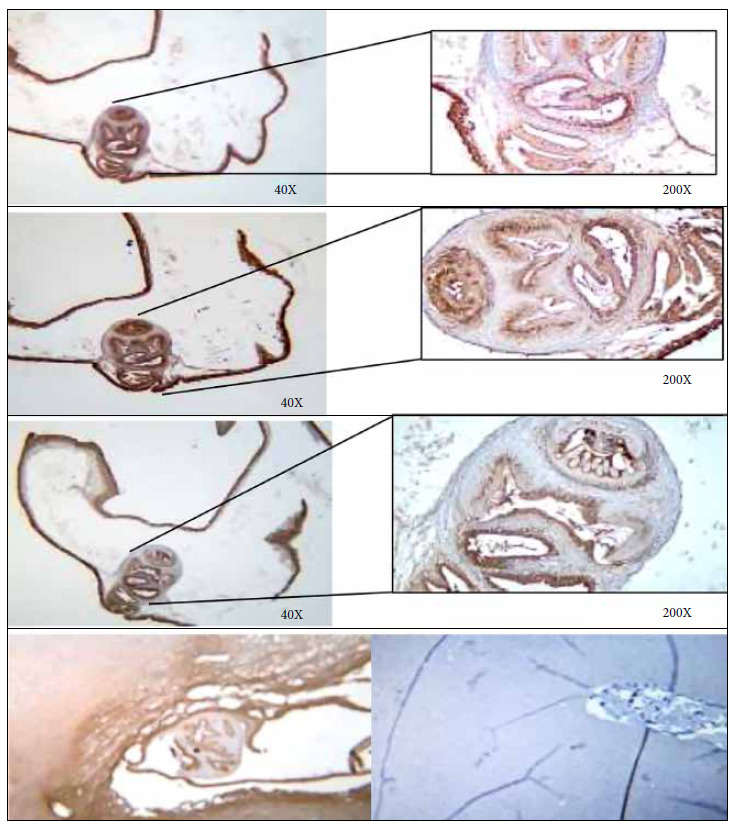
Immunohistochemistry of a cysticercus granuloma in sheep brain using the monoclonal antibodies TsW5 (top row), TsW11 (second row), and TsE3 (third row). Positive and negative controls (left to right) are shown in the bottom row.

Immunohistochemistry with the anti-*T solium* moAbs TsW5, Tsw11 and TsE3 was performed on the two granulomas found at necropsy corresponding to specimens #9092 and #9099. Macroscopically these two lesions were qualified as unspecific granulomas and immunohistochemistry confirmed the causative agent. One of them showed reactivity to the moAbs used, thus confirming that it was a cysticercus (Figures 3 and 4). In addition, microscopic evaluation of the same sample showed the presence of a characteristic scolex, which confirms that it was a *T. solium* cyst.

## DISCUSSION

This exploratory experiment provides proof of concept that experimental intracranial infection with *T. solium* posoncospheres can cause NCC in the sheep model. We found viable parasitic infection and at least one cysticercosal granuloma in two of the five sheep treated with methylprednisolone, suggesting that immunity may play an important role in this experimental model of infection. We used methylprednisolone, a glucocorticoid commonly used to treat inflammatory disorders and as an immunosuppressant in organ transplantation. White blood cells are the main target of this drug. A short three-day treatment schedule was used, similar to that used by Feltrin *et al*. in goats ^20^, which showed a significant decrease in WBC count from baseline and up to 28 days later. However, the dose used in this experiment was lower (1 mg/kg vs. 10 mg/kg) so it is possible that the sheep did not reach an adequate range of immunosuppression.

The viable ventricular cyst found in one of the sheep could be confirmed with the naked eye and microscopically by examining the stained slide. On the other hand, immunohistochemistry was used for nonspecific granulomas. Immunohistochemistry allows us to detect whether specific antigens are present and their microanatomic location, allowing us to identify the lineage of poorly differentiated tissue cell populations. In addition, this technique preserves the histologic architecture, which is not possible with other molecular methods. Only one of the two suspected lesions reacted to moAb. Although macroscopically we observed only an inflammatory nodule, histology and immunohistochemistry confirmed that it was a cysticercus.

The study of NCC in patients has provided important information about the disease, however, there are limitations, samples are collected using minimally invasive procedures, including blood samples, and brain tissue or cerebrospinal fluid samples, which can only be obtained for clinical or diagnostic need. In addition, there is a need to find better therapeutic options for patients with epilepsy secondary to NCC, as many patients suffer seizures refractory to available treatment ^21^. Therefore, the continued use and development of appropriate animal models for the study of this disease is necessary. Animal models using *T. solium*, *Taenia saginata*, *Taenia crassiceps* and *Mesocestoides corti* have been reported in mice, rats, sheep, pigs and even rhesus monkeys (*Macaca mulatta*) ^21^. However, experimental murine models using *T. solium* in recent years, despite showing a high infection rate in immunosuppressed mice, showed much variation in infection rates, cyst load, and antibody and antigen levels, with no obvious correlation between the number of cysticerci. Infection models in pigs also showed high variability, with cysticerci recovery rates from 0.2 to 81.93% and has been reported that the number of viable cysts decreased with increasing age, probably due to the presence of maternal antibodies, their future elimination could achieve higher infection rates ^22^. Advances in animal models have been reported; however, a model of intracranial infection with *T. solium* in sheep has not yet been described.

There is a great need for animal models of NCC that are capable of developing seizures and epilepsy. A more suitable animal model would represent a major step in the study of NCC-related epilepsy. The rat model has been used to study intracranial infection with NCC, which has some advantages such as easy handling, low maintenance cost, high availability, and surgical and experimental protocols ^13^. However, the rat model has two main limitations. First, rodents differ significantly from humans, both in size and neuroanatomical organization, which leads to questions regarding lesion size relative to the murine brain. Second, rats have a much shorter lifespan than humans, which is an impediment to studying chronic disease conditions, such as epilepsy.

On the other hand, the pig, a natural intermediate host of *T. solium*, has not been used as an epilepsy model for a number of reasons. It rarely has clinically detectable seizures and invasive procedures are difficult to perform because of the thickness of its skull. Almost no electroencephalography has been performed in pigs ^(23, 24)^. In addition, the maintenance and management of pigs is more demanding and costly than that of the ovine model ^25^. These drawbacks are reduced by using a model of NCC infection in young sheep. The average life expectancy of a sheep is 12 to 15 years (rat: 2-3 years) ^26^ ^,^ ^27^. The sheep brain has a cerebral cortex with more convolutions and structures similar to those of humans and a weight of 180 g (rat: 2 g, human: 1300-1400 g). Also, the availability of well-detailed atlases of the sheep brain makes interpretations, translational research, and stereotaxic intervention possible.

Sheep have been widely used as biomedical models for the investigation of various physiological and pathological conditions ^28^. Coenurosis neurological infection, the larval stage of *Taenia multiceps,* has been widely reported in sheep. Transmission of coenurosis is similar to *T. solium*, follows the fecal-oral route and the metacestode (larval stage) develops in the brain and spinal cord ^29^, demonstrating the ability of cestodes to infect central nervous system structures and develop brain cysts in sheep. Epilepsy-related studies in the sheep model consistently showed that this mammal is capable of developing epilepsy and status epilepticus. The sheep model has been used to study provoked seizures and to measure the electrical activity generated by epilepsy, which can be registered by electrodes on the skin, subcutaneously or within the cortical area by electroencephalography; likewise, the clinical manifestations of epilepsy can be observed in this animal model ^14^ ^,^ ^30^.

Our study is exploratory and has limitations. In order to improve the viability of the model, we highlight the need to use sheep free of other parasitic infections prior to the use of immunosuppressants to reduce the reactivation of infections, which may interfere with the experiment, serology or increase mortality in the study animals. The lack of exhaustive evaluation of the study animals was one of the major limitations of this study, as well as the used dose of immunosuppression, which was ten times lower than the recommended doses ^20^, with the possibility that the sheep did not reach an adequate range of immunosuppression.

It would be of interest to evaluate the infectivity of higher doses of posoncosphere inoculation, with animals acquired from industrialized farms to avoid other concomitant parasitosis, and to use a more aggressive immunosuppression regimen along with the use of immunity markers to demonstrate the response to corticosteroids.

In recent years, several animal models have been proposed in order to study cysticercosis in animals with different degrees of success. Despite the limitations described above, at least two animals were successfully infected, demonstrating that a sheep model of NCC is feasible.

This study provides proof of concept that experimental intracranial infection with *T. solium* posoncospheres can cause NCC in the sheep model. This could serve as a key step to study new therapy approaches, poorly understood aspects of this disease and the chronic effects of NCC and epilepsy on the brain.
