# Supplementary material for: Experimental brain infection with cysticercosis in sheep
Source: Rev Peru Med Exp Salud Publica. 2022 Sep 30;39(3):328–35. doi: 10.17843/rpmesp.2022.393.11039 (PMC9899549; doi:10.17843/rpmesp.2022.393.11039)
Supplement: Translation

# Translation: Infección experimental cerebral con cisticercosis en ovejas

**Authors:** Katherine A. Sota, Javier A. Bustos, Manuela R. Verastegui, Luz Toribio, Nancy Chile, Noelia Angulo, Carla Cangalaya, Juan Calcina, Armando E. González, Robert H. Gilman, Héctor H. García

**Affiliations:** 1 Unidad de Cisticercosis, Instituto Nacional de Ciencias Neurológicas, Lima, Perú.; 2 Centro de Salud Global, Universidad Peruana Cayetano Heredia, Lima, Perú.; 3 Laboratorios de Investigación y Desarrollo, Universidad Peruana Cayetano Heredia, Lima, Perú.; 4 Escuela de Medicina Veterinaria, Universidad Nacional Mayor de San Marcos, Lima, Perú.; 5 Department of International Health, Johns Hopkins Bloomberg School of Public Health, Baltimore, Maryland, US.

**Keywords:** Epilepsia, *Taenia solium*, Cisticercosis, Neurocisticercosis, Oveja

## Abstract

**Objetivo:**

. Explorar la viabilidad de desarrollar un modelo de neurocisticercosis (NCC) de oveja mediante infección intracraneal de oncosferas de *T. solium.*

**Materiales y métodos.:**

Se realizó un modelo de infección experimental de NCC en ovejas. Se inocularon aproximadamente 10 posoncósferas de *T. solium* cultivadas previamente por 30 días por vía intracraneal en diez ovejas. Las oncósferas, en 0,1 mL de solución salina fisiológica, se inyectaron en el lóbulo parietal a través de una aguja de calibre 18.

**Resultados.:**

Después de tres meses, en dos ovejas se encontraron granulomas y en una tercera identificó un quiste de 5 mm de diámetro en el ventrículo lateral derecho y la evaluación histológica confirmó que el quiste corresponde a una larva de *T. solium*. También se utilizó inmunohistoquímica con anticuerpos monoclonales dirigidos contra componentes de membrana y antígenos excretorios/secretorios del quiste de *T. solium *para confirmar la etiología de los granulomas encontrados. Uno de ellos mostro reactividad ante los anticuerpos monoclonales utilizados, confirmando así que se trató de un cisticerco.

**Conclusión.:**

Este experimento es la prueba de concepto de que es posible infectar ovejas con cisticercosis por inoculación intracraneal.

## INTRODUCCIÓN

La neurocisticercosis (NCC) es la infestación del sistema nervioso humano por la fase larvaria de la *T. solium.* Es un problema de salud pública importante en la mayoría de los países en vías de desarrollo, asociado con morbilidad neurológica significativa ^1^. El diagnóstico de esta enfermedad también está en aumento en países industrializados debido a la migración procedente de zonas endémicas, con aproximadamente 2000 nuevos casos diagnosticados por año solo en los Estados Unidos, donde las hospitalizaciones y los costos asociados atribuibles a NCC superan los totales de malaria, así como de todas las otras enfermedades tropicales desatendidas ^2^. En el Perú, y en la mayoría de las regiones endémicas, alrededor de 30% de todos los síndromes epilépticos parecen ser atribuibles a NCC ^3^.

Los seres humanos suelen infectarse con huevos de *T. solium* por la vía fecal-oral. En el intestino, el fluido intestinal disuelve la cubierta de los huevos, liberando y activando a la oncósfera. Una vez activada puede penetrar en la pared intestinal. Cuando llega al tejido a través del torrente sanguíneo, generalmente en el músculo o en el sistema nervioso central, la oncósfera se establece y se desarrolla el cisticerco. Posteriormente, el parásito produce una variedad de moléculas que modulan la respuesta inmune del huésped para evitar su destrucción ^4^. Las manifestaciones clínicas dependen de los órganos afectados. En los seres humanos los síntomas se deben principalmente a la afectación del sistema nervioso central. Los quistes situados dentro del parénquima cerebral (NCC intraparenquimatosa), por lo general se degeneran en la siguiente secuencia: de quistes viables a quistes inflamados, a tejido granulomatoso focal, y aproximadamente el 40% termina con la formación de una cicatriz calcificada ^5^.

Muchos aspectos de la enfermedad, tales como la inflamación, respuesta inmune, el proceso de degeneración parasitaria y calcificación, los mecanismos que conducen a las convulsiones y la epilepsia, y las funciones de tratamiento antiparasitario y antiinflamatoria, todavía están poco estudiados, en gran parte debido a la falta de modelos animales adecuados.

Se han utilizado cerdos naturalmente infectados para estudiar aspectos inmunológicos e histopatológicos ^6^ ^,^ ^7^, así como para evaluar la farmacocinética, seguridad y eficacia de tratamientos antiparasitarios ^8^ ^,^ ^9^. El modelo porcino ha servido también para estudios de infección experimental en esta enfermedad ^7^ ^,^ ^10^ ^,^ ^11^. No obstante, la infección experimental del sistema nervioso central (SNC) del cerdo con *T. solium* por vía oral es difícil debido a la variabilidad de la eficacia de la infección oral. Nuestro grupo ha desarrollado un modelo de inyección intracarotídea de oncósferas que produce NCC consistentemente ^12^. Sin embargo, el modelo porcino tiene como desventajas el costo de la compra y el mantenimiento de los cerdos durante un largo tiempo y, sobre todo, la escasez de reactivos comerciales disponibles para la detección de biomarcadores en cerdos ^13^. Adicionalmente, los cerdos rara vez presentan convulsiones clínicamente evidentes.

Nuestro grupo ha estandarizado también un modelo de infección experimental de cisticercosis en cerebro de ratas. En este modelo se inyectaron intracranealmente oncósferas activadas de *T. solium *las cuales desarrollaron en quistes cerebrales o metacéstodos con características idénticas a las observadas en el huésped natural. Aunque este modelo no pasa por la ruta intestinal habitual de entrada, se produjeron infecciones reproducibles similares a las observadas en cerdos y seres humanos. La mayoría de las ratas infectadas (64%, n=42) desarrollaron quistes de cisticerco en sus cerebros. La infección fue exitosa en 83% (10/12) de ratas inyectadas con 500 o 750 oncósferas activadas, produciendo ratas con uno o más quistes. Además, las ratas infectadas desarrollaron anticuerpos específicos para *T. solium*, y se pudo también detectar antígeno circulante para *T. solium *en suero y líquido cefalorraquídeo (LCR), utilizando el ensayo por inmunoabsorción ligado a enzimas (ELISA). Este mismo modelo se ha utilizado inyectando la forma posoncósfera, ya cultivada *in vitro* por 15 días. En el 2019, Palma *et al. *infectaron ocho ratas Holtzman intracranealmente con diez posoncósferas de *T. solium*. Cuatro meses después se observó que un 63% (n=5) de estas presentaron infección intracraneal con *T. solium* ^4^. Sin embargo, estos modelos presentan limitaciones. La rata no es el huésped natural de la enfermedad pues tiene poco tiempo de vida, lo cual no permite estudiar cronicidad de la enfermedad y, debido al tamaño de su cerebro, podría haber una sobreestimación del efecto de masa y la inflamación causada por el quiste de *T. solium* ^13^.

Las ovejas han sido utilizadas como modelo animal para varios propósitos en enfermedades neurológicas, incluyendo la epilepsia, y no neurológicas. El modelo ovino se considera un modelo animal adecuado para estudiar epilepsia debido a su tamaño, disponibilidad y bajo costo de mantenimiento. Además, su disposición anatómica facilita la aplicación de técnicas estándar de neurocirugía y neurorradiología aplicada en humanos, así como el uso de resonancia magnética y otros métodos de diagnóstico por imágenes ^14^ ^,^ ^15^.

Este estudio tomó ventaja de los estudios realizados para estandarizar el modelo de infección intracerebral en ratas con el objetivo de explorar la viabilidad de desarrollar un modelo de infección experimental con NCC en ovejas por medio de la infección intracraneal con posoncósferas de *T. solium.*

**MENSAJE CLAVE**

Motivación para realizar el estudio: un modelo animal apropiado es necesario para estudiar aspectos poco conocidos de la NCC y epilepsia, como nuevos tratamientos antiepilépticos, vacunas, la respuesta inflamatoria cerebral y su manejo, así como los procesos de calcificación y epileptogénesis.

Principales hallazgos: se encontró granulomas en dos ovejas y se identificó un quiste en el ventrículo lateral derecho en una tercera.

Implicancias: este experimento es la prueba de concepto de que es posible infectar ovejas con cisticercosis por inoculación intracraneal, y un paso inicial para establecer un nuevo modelo animal capaz de estudiar nuevos enfoques de esta enfermedad y los efectos crónicos de la NCC y la epilepsia.

## MATERIALES Y MÉTODOS

### Animales

Se infectaron diez ovejas sanas de entre cuatro y ocho meses de edad, adquiridas de granjas rurales no industrializadas. Los animales fueron revisados por un veterinario para confirmar su buen estado de salud y transportadas a las instalaciones para animales mayores de la Escuela de Medicina Veterinaria de la Universidad Nacional Mayor de San Marcos en Lima. Las ovejas fueron alojadas en un corral de 5x5 metros y mantenidas en ciclos de 12/12 horas de luz/oscuridad con una temperatura media controlada de 16-18 ºC, sal mineralizada y alimento en raciones determinadas por edad y agua *ad libitum*.

### Aspectos éticos

El protocolo se llevó a cabo bajo la aprobación del Comité Institucional de Ética para el Cuidado y Uso de Animales de la Universidad Peruana Cayetano Heredia.

### Preparación de las posoncósferas

En base a los protocolos previos para infección experimental en cerebro de rata ^13^ se realizó una infección experimental usando posoncósferas de *T. solium*. Para obtener la etapa de posoncósfera se obtuvieron huevos de proglótides grávidas de *T. solium* expulsadas luego de tratamiento estándar de portadores de tenia con niclosamida ^13^ y se incubaron en hipoclorito de sodio (0,75%) durante 10 min a 4 °C. Posteriormente, las oncósferas fueron activadas para luego crecer en una monocapa de células de cultivo HCT-8 durante treinta días ^13^ ^,^ ^16^.

*Infección intracraneal*

Se inocularon aproximadamente diez posoncósferas de *T. solium* por vía intracraneal a cada oveja. Los animales fueron anestesiados y sedados usando ketamina (Ket-A-10®), 20 mg/kg intramuscular (IM); clorhidrato de xilazina (Dormi-Xyl®2), 0,3 mg/kg IM, y ketoprofeno (Profenid®) 3 mg/kg IM. En condiciones de asepsia adecuada se realizó una incisión de 2 cm en la piel del área parietal derecha, y luego se efectuó un orificio en la superficie ósea con un taladro de 2 mm de diámetro (Figura 1). Las posoncósferas en 0,1 mL de solución salina fisiológica se inyectaron en el lóbulo parietal con una aguja de calibre 18. La aguja de calibre 18 tiene un diámetro interior de 0,84 mm lo que permite el paso de oncósferas de 30 días de maduración, las cuales tienen un diámetro de 0,5 mm, aproximadamente. La aguja se introdujo aproximadamente a 2 cm de profundidad para llegar al parénquima cerebral parietal. Cinco de los diez animales fueron inmunosuprimidos usando 1 mg/kg/día de metilprednisolona por 3 días, inmediatamente después de la infección ^17^. Además, recibieron como terapia analgésica posquirúrgica ketoprofeno 2 mg/kg/día IM durante 3 días.

**Figura 1:** Procedimiento para hacer un pequeño orificio sobre el lóbulo parietal derecho.

Los animales fueron eutanizados bajo anestesia general, 3 meses después de la infección experimental. Se utilizó una combinación intramuscular de ketamina (20 mg/kg), y clorhidrato de xilazina (2 mg/kg), vía IM. Posteriormente, se utilizó una sobredosis de pentobarbital sódico como hipnótico y anticonvulsivante (Halatal®) 120 mg/kg, también vía IM. La totalidad de los cerebros fueron retirados para examen y disección. Las estructuras anómalas fueron identificadas y teñidas con hematoxilina-eosina (H-E) para su análisis. Además, los órganos internos fueron examinados cuidadosamente para identificar infecciones parasitarias concomitantes.

*Inmunohistoquímica*

Se utilizo inmunohistoquímica (IHC) con moAbs dirigidos contra componentes de membrana y antígenos excretorios/secretorios del quiste de *T solium* para confirmar la etiología de las lesiones encontradas ^18^.

Las lesiones sospechosas rodeadas de tejido se colocaron en casetes que fueron rotulados y sumergidos en *buffer* paraformaldehído al 10%, luego fueron sumergidos en etanol al 70% durante 24 a 48 h y, finalmente, fue incluido en parafina de acuerdo a los procedimientos estándar. Las secciones de tejido se adhirieron a láminas portaobjetos impregnadas con polilisina, que fueron desparafinadas y rehidratadas por inmersión en xilol, etanol (absoluto, 96% y 70%). La IHC se realizó inactivando la peroxidasa endógena con una dilución (1:5) de H_2_O_2_ durante 30 min a temperatura ambiente y en oscuridad. Para la recuperación del antígeno se utilizó una solución de citrato a 95 °C durante 8 min y se dejó enfriar. Luego, las secciones de tejido se lavaron tres veces con solución tamponada de PBS (pH 7,2) y se agregó la solución de bloqueo con 10% de suero de cabra y 6% de leche diluida en PBS-*Tween* 0,05% -Tritón 0,1% durante 1 h. Los sobrenadantes de cultivo de moAbs anti-*T solium* TsW5/ Tsw11/ TsE3 ^18^. Se diluyeron a la concentración determinada en PBS-*Tween* 0,05% -Tritón 0,1% y se incubaron a 4 °C durante toda la noche. Adicionalmente, colocamos controles positivos (quistes de *T solium* provenientes de cerdos), negativos (tejido cerebral de oveja sana) y de técnica (quistes de oveja y cerdo sin agregar el moAb). Las láminas se lavaron tres veces con PBS-Tween 0,05% y se agregó al anticuerpo secundario antirratón IgG conjugado con biotinilado diluido 1/700 en solución bloqueante con 10% de suero de cerdo y se incubó por 30 min. Las uniones de moAbs-antígenos fueron amplificadas con streptavidina-HRP durante 30 min. Finalmente, la reacción se evidenció utilizando una solución con el cromógeno 3,3-diaminobencidina (DAB); se agregó la solución de hematoxilina como contraste de tejido, seguido de la deshidratación con alcoholes y xilol, y se realizó el montaje.

## RESULTADOS

La inoculación intracraneal con posoncósferas se realizó en diez ovejas. No hubo complicaciones después de la intervención y los animales no mostraron signos de dolor según la escala propuesta por Guedes *et al.* ^19^. Al cabo de una hora después de suministrar la anestesia, las ovejas volvieron gradualmente a su comportamiento normal. Tres ovejas murieron antes del final de los 90 días de seguimiento. Por informes de necropsia, dos de ellas fallecieron por obstrucción intestinal por *Moniezia expansa,* y otra debido a una infección bacteriana intestinal. Las siete ovejas restantes, cinco del grupo inmunosuprimido y dos del grupo no inmunosuprimido, fueron sacrificadas 3 meses después de la inoculación. Los cerebros se extrajeron en su integridad para el análisis patológico. En cuatro de ellos no se encontró ninguna estructura que sugiera infección por cisticercosis, en dos ovejas se encontraron lesiones granulomatosas no específicas y en una se identificó un quiste ventricular protruido de 5 mm de diámetro en el ventrículo lateral derecho. Por observación microscópica directa y evaluación histológica con tinción de H-E se confirmó que el quiste corresponde a una larva de *T. solium*, un rostelo (corona de ganchos) y cuatro ventosas fueron identificados claramente (Figura 2). La microscopía de uno de los granulomas también demostró remanentes de tejido parasitario compatible con una larva de céstodo, y el otro mostró solamente un conglomerado de linfocitos. Estos hallazgos anormales (dos granulomas y un quiste) fueron encontrados en tres ovejas pertenecientes al grupo inmunosuprimido.

**Figura 2:** A-B: fotografía del cerebro de una oveja que muestra un quiste ventricular (flecha negra). C: Quiste ventricular con escólex evaginado. D: Tinción de H-E (40x) muestra escólex, cuatro ventosas y un rostelo.

Todas las ovejas mostraron otras infecciones parasitarias concomitantes en la necropsia. Ocho estaban infectadas con *Moniezia expansa*, cinco con *Fasciola hepatica*, dos con *Thysanosoma actinioides* y dos con *Taenia hydatigena*. Seis de ellas tenían infecciones multiparasitarias.

*Inmunohistoquímica*

Se realizó inmunohistoquímica con los moAbs anti-*T solium* TsW5, Tsw11 y TsE3 a los dos granulomas encontrados en la necropsia correspondientes a los especímenes #9092 y #9099. Macroscópicamente estas dos lesiones fueron calificadas como granulomas inespecíficos por lo que se utilizó inmunohistoquímica con el propósito de confirmar el agente causante. Una de ellas mostro reactividad ante los moAbs utilizados, confirmando así que se trata de un cisticerco (Figuras 3 y 4). Además, la evaluación microscópica de la misma muestra presencia de un escólex característico, lo cual confirma que se trataría de un quiste de *T. solium.*

**Figura 3:** Fotografía del cerebro de una oveja que muestra un granuloma no específico.

**Figura 4:** Inmunohistoquímica de un granuloma cisticercoso en el cerebro de una oveja usando los anticuerpos monoclonales TsW5 (fila superior), TsW11 (segunda fila), y TsE3 (tercera fila). En la fila inferior se muestran los controles positivo y negativo (izquierda a derecha).

## DISCUSIÓN

Este experimento exploratorio proporciona la prueba de concepto de que la infección intracraneal experimental con posoncósferas de T. solium pueden causar NCC en el modelo de oveja. Encontramos una infección parasitaria viable y al menos un granuloma cisticercoso en dos de las cinco ovejas tratadas con metilprednisolona, lo que sugiere que la inmunidad puede cumplir una función importante en este modelo experimental de infección. Se utilizó metilprednisolona, un glucocorticoide comúnmente utilizado para tratar trastornos inflamatorios y como inmunosupresor en trasplantes de órganos. El principal objetivo de este fármaco son los glóbulos blancos. Se utilizó un esquema corto de tratamiento, por tres días, similar al utilizado por Feltrin *et al.* en cabras ^20^, el cual demostró disminución significativa del conteo de glóbulos blancos con respecto al valor basal y hasta a los 28 días posteriores. Sin embargo, la dosis utilizada en este experimento fue inferior en dosis (1 mg/kg vs. 10 mg/kg) por lo que es posible que las ovejas no llegaran a un rango de inmunosupresión adecuada. 

El quiste ventricular viable encontrado en una de las ovejas pudo confirmarse a simple vista y microscópicamente examinando la lámina portaobjetos teñida. Por otro lado, para los granulomas inespecíficos se utilizó inmunohistoquímica. La inmunohistoquímica nos permite detectar si hay antígenos específicos presentes y su ubicación microanatómica, permitiendo identificar el linaje de las poblaciones celulares de tejido pobremente diferenciado. Además, esta técnica preserva la arquitectura histológica, lo cual no es posible con otros métodos moleculares. Solo una de las dos lesiones sospechosas reaccionó a los moAb. Si bien macroscópicamente solo se observaba un nódulo inflamatorio, la histología y la inmunohistoquímica confirmaron que se trataba de un cisticerco.

El estudio de NCC en pacientes ha proporcionado información importante sobre la enfermedad, sin embargo, existen limitaciones, las muestras se recolectan usando procedimientos mínimamente invasivos, entre ellos muestras de sangre, y las muestras de tejido cerebral o líquido cefalorraquídeo, las cuales solo se pueden obtener por necesidad clínica o diagnóstica. Además, es necesario encontrar mejores opciones terapéuticas para pacientes con epilepsia secundaria a NCC, ya que muchos pacientes sufren convulsiones refractarias al tratamiento disponible ^21^. Por esto, es necesario el uso y desarrollo continuo de modelos animales apropiados para el estudio de esta enfermedad. Se han reportado modelos animales que utilizan *T. solium*, *Taenia saginata, Taenia crassiceps* y *Mesocestoides corti* en ratones, ratas, ovejas, cerdos e incluso monos Rhesus (*Macaca mulatta*) ^21^. Sin embargo, los modelos murinos experimentales utilizando *T. solium *en los últimos años, pese a mostrar una alta tasa de infección en ratones inmunodeprimidos, presentaron mucha variación en las tasas de infección, carga de quistes y los niveles de anticuerpos y antígenos, sin una correlación evidente entre el número de cisticercos. Los modelos de infección en cerdos también mostraron elevada variabilidad, con tasas de recuperación de cisticercos de 0,2 a 81,93% y se observó que el número de quistes viables se redujo con el aumento de la edad, probablemente por la presencia de anticuerpos maternos, su futura eliminación podría lograr tasas más altas de infección ^22^. Se han descrito avances en los modelos animales, sin embargo, un modelo de infección intracraneal con T. solium en ovejas no ha sido aún descrito.

Existe una gran necesidad de modelos animales de NCC que sean capaces de desarrollar convulsiones y epilepsia. Un modelo animal más adecuado representaría un gran paso para el estudio de la epilepsia relacionada a la NCC. Se ha utilizado el modelo de rata para estudiar infección intracraneal con NCC, el cual tiene algunas ventajas como su fácil manejo, bajo costo de mantenimiento, alta disponibilidad y protocolos quirúrgicos y experimentales ^13^. Sin embargo, el modelo de rata tiene dos limitaciones principales. En primer lugar, los roedores difieren significativamente de los seres humanos, tanto en tamaño como en su organización neuroanatómica, lo cual lleva a cuestionamientos en cuanto al tamaño de la lesión en relación al cerebro murino. En segundo lugar, las ratas tienen una vida mucho más corta que los seres humanos, lo cual es un impedimento para estudiar condiciones crónicas de la enfermedad, como la epilepsia. 

Por otro lado, el cerdo, huésped intermediario natural de la *T. solium,* no se ha desarrollado como modelo de epilepsia por un número de razones. Rara vez tiene convulsiones clínicamente detectables y los procedimientos invasivos son difíciles de realizar debido al espesor de su cráneo. Casi no se ha realizado electroencefalografía en cerdos ^23^ ^,^ ^24^. Además, el mantenimiento y manejo de los cerdos es más exigente y costoso que el del modelo ovino ^25^. Estos inconvenientes se reducen utilizando un modelo de infección por NCC en ovejas jóvenes. La media de la esperanza de vida de una oveja es de 12 a 15 años (rata: 2-3 años) ^26^ ^,^ ^27^. El cerebro ovino tiene una corteza cerebral con más circunvoluciones y estructuras similares a las del ser humano y un peso de 180 g (rata: 2 g, humano: 1300-1400 g)). También la disponibilidad de Atlas bien detallados del cerebro de ovejas hace que las interpretaciones, la investigación traslacional y la intervención estereotáxica sean posibles. 

Las ovejas se han utilizado ampliamente como modelos biomédicos para la investigación de varias condiciones fisiológicas y patológicas ^28^. La infección neurológica por cenurosis, la fase larvaria de *Taenia multiceps* ha sido ampliamente reportada en el ganado ovino. La transmisión de cenurosis es similar a *T. solium,* sigue la ruta fecal-oral y el metacéstodo (fase larvaria) se desarrolla en el cerebro y la médula espinal ^29^, demostrando la capacidad de los cestodos para infectar estructuras del sistema nervioso central y desarrollar quistes cerebrales en el ganado ovino. Los estudios relacionados con epilepsia en el modelo ovino mostraron consistentemente que este mamífero es capaz de desarrollar epilepsia y estatus epiléptico. El modelo ovino ha servido para estudiar convulsiones provocadas y medir la actividad eléctrica generada por la epilepsia, la cual puede ser registrada por electrodos sobre la piel, por vía subcutánea o dentro del área cortical por medio de electroencefalografía; asimismo, la manifestación clínica de la epilepsia se puede observar en este modelo animal ^14^ ^,^ ^30^.

Nuestro estudio es exploratorio y con limitaciones. Con el fin de mejorar la viabilidad del modelo, se resalta la necesidad de utilizar ovejas libres de otras infecciones parasitarias previo al uso de inmunosupresores para disminuir la reactivación de infecciones, que puedan interferir con el experimento, serología o aumenten la mortalidad en los animales de estudio. La falta de evaluación exhaustiva de los animales de estudio fue una de las mayores limitaciones de este estudio, así como la dosis utilizada de inmunosupresión, la que fue diez veces inferior a las dosis recomendadas ^20^, existiendo la posibilidad de que las ovejas no llegaran a un rango de inmunosupresión adecuada. 

Seria de interés evaluar la infectividad de dosis mayores de inoculación de posoncósferas, con animales adquiridos de en criaderos industrializados para evitar así otras parasitosis concomitantes, y utilizar un régimen de inmunosupresión más agresiva acompañado del uso de marcadores de inmunidad para evidenciar la respuesta a los corticoides. 

En los últimos años se han propuesto diversos modelos animales para estudiar cisticercosis en animales demostrando diversos grados de éxito. A pesar de las limitaciones descritas líneas arriba, al menos dos animales se lograron infectar, demostrando que es factible llegar a un modelo ovino de NCC.

Este estudio proporciona la prueba de concepto de que la infección intracraneal experimental con posoncósferas de *T. solium* pueden causar NCC en el modelo de oveja. Esto podría servir como paso clave para estudiar nuevos enfoques de terapia, aspectos poco conocidos de esta enfermedad y los efectos crónicos de la NCC y la epilepsia en el cerebro.
